# A Psychosis Superspectrum in Borderline Personality Disorder?

**DOI:** 10.1192/j.eurpsy.2023.426

**Published:** 2023-07-19

**Authors:** J. Henriques-Calado, M. Paulino, J. Gama Marques

**Affiliations:** ^1^Faculdade de Psicologia, Universidade de Lisboa, Alameda da Universidade, 1649-013 Lisboa, Portugal; ^2^CICPSI, Faculdade de Psicologia, Universidade de Lisboa, Alameda da Universidade, 1649-013 Lisboa, Portugal; ^3^Clínica Universitária de Psiquiatra e Psicologia Médica, Faculdade de Medicina, Universidade de Lisboa, Avenida Professor Egas Moniz, 1649-028 Lisboa, Portugal; ^4^Consulta de Esquizofrenia Resistente, Hospital Júlio de Matos, Centro Hospitalar Psiquiátrico de Lisboa, Avenida do Brasil, 53, 1749-002 Lisboa, Portugal

## Abstract

**Introduction:**

The innermost relationship of the borderline concept and psychosis has been historically intertwined and can be traced back to the 20th century, but remarkably, to date, they have not been the focus of many empirical studies. Likewise, the contributions of empirical research on the DSM-5 dimensional approach to this topic are also uncommon.

**Objectives:**

In this study the framework of psychosis superspectrum were put closely in relation to both DSM-5 psychoticism/detachment domains, personality traits and psychopathological symptoms features in borderline personality disorder (PD).

**Methods:**

A cross-sectional study of a borderline PD sample of 58 participants (*M*_age_=39.76 years, *SD*=11.37; *M*_schooling_=9 years), mainly male (58.5%). Self-reported assessment: PID-5; BSI; NEO-FFI. A multiple linear regression was computed.

**Results:**

In borderline PD, the PID-5 disinhibition (β=.51), BSI psychoticism (β=.43), BSI depression (β=-.24) and NEO neuroticism (β=.29) predicted psychosis superspectrum, explaining 94% of the variance. Also, stands out as a complement that, the BSI psychoticism was predicted by PID-5 detachment and PID-5 psychoticism, explaining 82% of the variance.

**Conclusions:**

Evidence appears to be emerging for the underlying psychosis superspectrum trough borderline PD. There is a closer dialogue between the state-of-art view of a dimensional pathological personality-symptoms and the borderline pathology.

**Disclosure of Interest:**

None Declared

